# In-plane anisotropy and twin boundary effects in vanadium nitride under nanoindentation

**DOI:** 10.1038/s41598-017-05062-0

**Published:** 2017-07-06

**Authors:** Tao Fu, Xianghe Peng, Cheng Huang, Henggao Xiang, Shayuan Weng, Zhongchang Wang, Ning Hu

**Affiliations:** 1 College of Aerospace Engineering, Chongqing University, Chongqing, 400044 China; 2 State Key Laboratory of Coal Mine Disaster Dynamics and Control, Chongqing University, Chongqing, 400044 China; 3 International Iberian Nanotechnology Laboratory (INL), Avenida Mestre Jose Veiga, Braga, 4715-330 Portugal

## Abstract

Twin boundaries (TBs) have been observed in and introduced into nonmetallic materials in recent years, which brought new concepts for the design of new structural materials. However, the roles of TB on the mechanical properties and strengthening/softening of transition metal nitrides remain unclear. To investigate the TB effects and the in-plane anisotropy, nanoindentations on VN (111) films with and without TB were simulated with molecular dynamics, in which a cylindrical indenter was used, and its longitudinal axis were assigned along <112> and <110>, respectively. We found that the effect of the indenter orientation is insignificant in the elastic stage, but significant in the following inelastic deformation. Different deformation mechanisms can be found for inelastic deformation, such as twinning and dislocation glide. The migration of TB can be observed, which may release the internal stress, resulting in softening; while the dislocation locking and pileup at TB can enhance the strength. We also found that the strengthening/softening induced by TB depends on the deformation mechanisms induced by indenter directions.

## Introduction

Transition metal nitrides (TMNs), such as vanadium nitride (VN) and titanium nitride (TiN), have attracted increasing attention due to their excellent physical and mechanical properties, especially high hardness and wear resistance^1–4^. The testing of these mechanical properties usually involves plastic deformation. It is well known that twinning^5^ and dislocation glide^6^ are two of the most common mechanisms for plastic deformation. Twin boundary (TB), a symmetrical plane between the twin and the original parent lattice, plays a very important role in the mechanical properties, such as strength^7, 8^, hardness^9, 10^ and toughness^11^. While twinning may or may not contribute to overall plastic deformation, depending on specific twinning mechanisms and the reactions between twinning and other defects, it does result in substantial evolution of microstructures and the formation of TBs^12^. Li *et al*. found that there is a critical twin thickness, at which the yield strength reaches the maximum^7^, and proposed a dislocation nucleation governed softening mechanism. However, Zhou *et al*.^13^ and Zhu *et al*.^14^ found that, when subjected to an external stress parallel to the twin planes, the strength of metallic multilayers can be sustainably improved even though the twin spacing falls below a critical twin thickness, and proposed a jogged dislocation governed strengthening mechanism. Different deformation mechanisms may play different major roles under different stress states. At present, most work about TB focused mainly on metals, but less progress has been achieved in other kinds of materials, for example, ceramics materials, probably because those less experimental evidences have been reported in the literature.

In recent years, TBs have been observed in or introduced into nonmetallic materials, which brought new concepts for the design of new structural materials. For example, by introducing the nanotwin structures into cubic BN^9^ and diamond^10^ can significantly enhance their hardness. It may inspire people to improve the mechanical properties of ceramics by introducing nanotwin structures into rock-salt structure TMNs. Xue *et al*.^15^ identified {111} twin boundary in rock-salt MnS with a combined study of transmission electron microscopy (TEM) and density functional theory (DFT) calculation. It implies the possibility to introduce the twin structures into the rack-salt structure VN to improve its mechanical properties. Li *et al*.^16^ found that twin structure can be stable in rack-salt metal nitrides by analyzing the twin boundary energy. Yadav *et al*. and Fu *et al*. predicted the existence of {111} <112> partial dislocations (PDs) in TiN^17^ and VN^18^, respectively, based on the calculation of generalized stacking fault curves, which was thought to be the premise for twin nucleation. Fu *et al*.^19^ further found in MD simulations that deformation twin can form in VN under nanoindentation. However, the roles of TB on the mechanical properties and strengthening/softening effects in VN remain unclear.

It is still challenging to achieve quantitative results of the effects of TBs in VN in experiment. MD simulation may provide a powerful means to explore the effects of TB in the mechanical properties of nanocrystalline structural materials^7, 20^. It is known that the deformation mechanisms are different if a material is subjected to nanoindentation on different surfaces, for instance, VN (111)^19^ and (001)^21^, ascribed to the inherent anisotropy of monocrystalline rock-salt VN. On the other hand, etched W wire^22^ or wedge indenter^2^ was often used to perform nanoindentations, which can be equivalent to a two-dimensional (2D) cylindrical indenter. Therefore, 2D cylindrical indenters were usually used in MD nanoindentation simulations to investigate the mechanical responses^23–27^. However, the in-plane anisotropy and the corresponding deformation mechanisms may exist under nanoindentation if a cylindrical indenter is used, because the (111) surface has a three-fold rotation symmetry about its normal direction. In this work, we first investigate the in-plane anisotropy of VN (111) under nanoindentation with a cylindrical indenter along different axial directions, and then explore the effects of TB on the mechanical properties of VN.

## Calculation Methods

The modified embedded atom method (MEAM) potential developed by Baskes *et al*.^28–30^, which has been successfully applied to the tension^31^, deposition^32^, surface energy^33, 34^ and stacking energy^35, 36^, is chosen to express the interaction between the film atoms. The detailed parameters for the single element (V-V and N-N) potentials have been given by Baskes *et al*.^29^ and Lee *et al*.^30^, as listed in Table 1. The binary MEAM potential developed in term of the single element was used to describe the force between V and N^37^, the corresponding parameters are listed in Table 2. These potentials can not only reproduce the basic mechanical properties of V-N system, but also explore the fracture behaviors of VN layers^37^, the slip systems and deformation mechanism in VN under nanoindentation, incorporating generalized stacking fault energy curves^21, 38^. The pair Lennard-Jones potential, which was widely used to describe the contact at nano-scale^39^, is chosen to describe the interaction between the indenter and film. The parameters *ε* and *σ* are set to be 3.14 meV and 3.7588 Å for C-V^21, 40^, and 3.722 meV and 3.33 Å for C-N^41^, respectively. On the purpose to investigate in-plane anisotropy of VN (111) and the effects of TB, the indenter is treated as a rigid body. Therefore, as in some of our previous works^19, 21^, the interaction between the indenter atoms need not be involved in the simulation to save computational resources. Before the MD nanoindentation simulations, we calculate the GSFE and TFE curves, as shown in Figure S1 of Supplementary Materials.

**Table 1:** Parameters in MEAM potentials for pure V and N. *E*_*c*_, *r*_*e*_, *α*, *A*, *β*, *t*, *C*, and *d* denote cohesive energy, equilibrium nearest-neighbor distance, exponential decay factor, scaling factor for embedding energy, exponential decay factor, weigh factor for atomic densities, screening parameter, and adjustable parameter, respectively.

	*E*_*c*_(eV)	*r*_*e*_(Å)	*α*	_*A*_	*β* ^(0)^	*β* ^(1)^	*β* ^(2)^	*β* ^(3)^	*t* ^(0)^	*t* ^(1)^	*t* ^(2)^	*t* ^(3)^	*C*_min_	*C*_max_	*d*
V	5.30	2.625	4.81	0.73	4.74	1.0	2.5	1.0	1.0	3.30	3.2	−2.0	0.49	2.8	0
N	4.88	1.100	5.96	1.80	2.75	4.0	4.0	4.0	1.0	0.05	1.0	0.0	2.00	2.8	0

**Table 2:** A set of 2NN MEAM potential parameters for V-N system. *E*_*c*_, *r*_*e*_, *B*, *d*, and *C* represent cohesive energy, equilibrium distance, bulk modulus, adjustable parameter and screening parameter of B1 phase VN.

*E*_*c*_ (eV)	*r*_*e*_ (Å)	_*B*_ (GPa)	_*d*_	V*–*N*–*V		N*–*V*–*N		V*–*V*–*N		V*–*N*–*N	
				*C*_min_	*C*_max_	*C*_min_	*C*_max_	*C*_min_	*C*_max_	*C*_min_	*C*_max_
6.72	2.06	315	0	0.45	2.8	0.85	2.8	1.117	2.8	0.80	2.8

**Figure 1:**
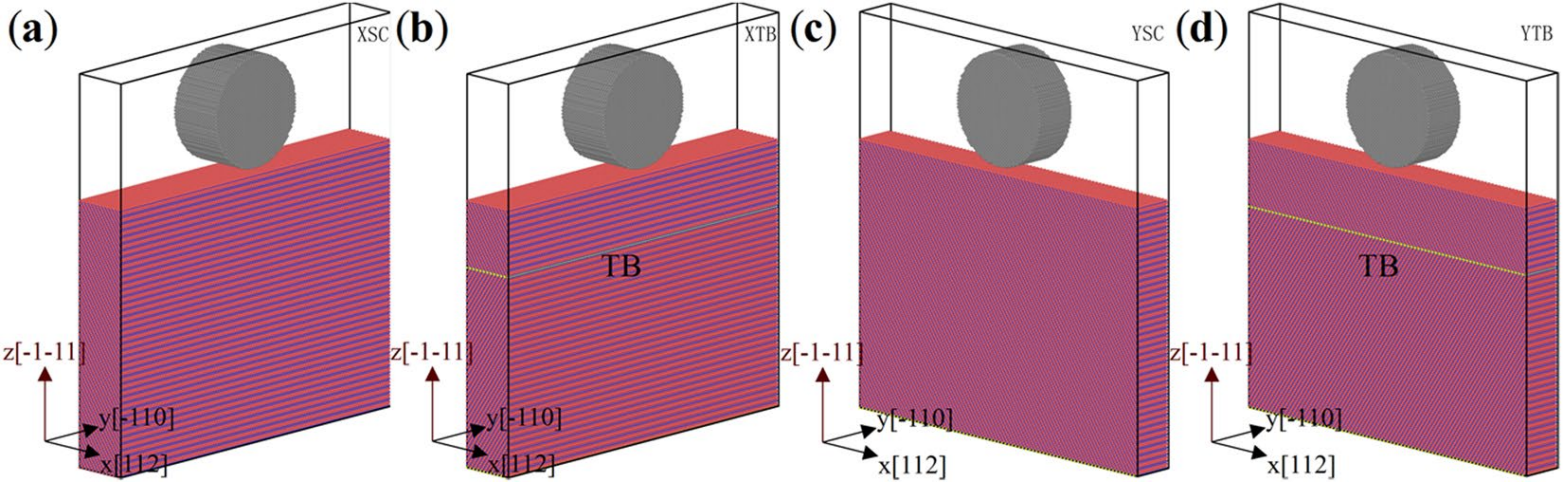
Atomic models for MD simulation of indentation. (**a**) XSC; (**b**) XTB; (**c**) YSC; (**d**) YTB. Red, blue and grey balls represent V, N and C atoms, respectively. Yellow atomic layer represents the twin boundary (TB).

In this work, four samples, named as XSC, XTB, YSC and YTB, are prepared to perform the MD nanoindentation simulations as shown in Fig. 1, the sizes and orientations of which are listed in Table 3. X and Y mean that the indenter longitudinal axis are parallel with different crystallographic directions *x* <112> and *y* <110>, and SC and TB represent single crystal and twin boundary, respectively. The *x*, *y* and *z* axes of the XSC, YSC, and the upper part of XTB and YTB films corresponded to [112], $[\bar{1}10]$ and $[\bar{1}\bar{1}1]$ crystal orientations, respectively, while that of the lower part of XTB and YTB films corresponded to $[\bar{1}\bar{1}\bar{2}]$, $[1\bar{1}0]$ and $[\bar{1}\bar{1}1]$ crystal orientations to form a TB. The thicknesses of the upper films of both the samples XTB and YTB are $10\sqrt{3}a$. The conjugate gradient (CG) algorithm is used at first to optimize the samples and make them reach stable configurations with minimum equilibrium energy. All simulations are performed at 10 K. The bottommost four layers of atoms are fixed to prevent the film from shifting, and the rest atoms are kept at a constant temperature of 10 K with the Langevin thermostat^42^ as the thermostat atoms during loading stage. The radius of the cylindrical diamond indenter is 60 Å and the indenter moves downwards at a constant speed of 40 m/s during indentation. Periodic boundary conditions are imposed in both *x* and *y* directions.

**Table 3:** Sizes and orientations of samples XSC, XTB, YSC and YTB. X and Y represent axis direction of cylindrical indenter. SC and TB represent single crystal and twin boundary, respectively. *a* = 4.12 Å is lattice constant.

Sample		*lx*	*ly*	*lz*
XSC		6 [112] *a*	70 $[\bar{1}10]$ *a*	40 $[\bar{1}\bar{1}1]$ *a*
XTB	upper	6 [112] *a*	70 $[\bar{1}10]$ *a*	10 $[\bar{1}\bar{1}1]$ *a*
	lower	6 $[\bar{1}\bar{1}\bar{2}]$ *a*	70 $[1\bar{1}0]$ *a*	30 $[\bar{1}\bar{1}1]$ *a*
YSC		40 [112] *a*	8 $[\bar{1}10]$ *a*	40 $[\bar{1}\bar{1}1]$ *a*
YTB	upper	40 [112] *a*	8 $[\bar{1}10]$ *a*	10 $[\bar{1}\bar{1}1]$ *a*
	lower	40 $[\bar{1}\bar{1}\bar{2}]$ *a*	8 $[1\bar{1}0]$ *a*	30 $[\bar{1}\bar{1}1]$ *a*

Centro-symmetry parameter (*CSP*), which can be used to describe the local disorder, was originally developed for BCC and FCC lattice structures. It can also be applied to a diamond cubic lattice and NaCl structure by considering them as nested structure with two identical FCC lattices^37, 43^. The *CSP* for each atom are calculated to analyze the microstructure with the following formula^44^,

1 $$CSP={\sum _{i=1}^{N/2}|{{\bf{R}}}_{i}+{{\bf{R}}}_{i+N/2}|}^{2},$$

where *N* = 6 is the nearest coordination number of a central atom in NaCl (B1) structure VN, **R**_*i*_ and **R**_*i*+*N*/2_ are the vectors from the central atom to a particular pair of the nearest neighbors, respectively. The parameter is zero for each atom whose neighboring atoms are located at the site of the perfect lattice structure. If there is defect such as vacancy and dislocation, this parameter for an atom in the vicinity of the defect will become much larger than that just caused by the local atomic vibration. We use the open software, OVITO^45^, for visualization analysis.

## Results and Discussion

### Elastic deformation

**Figure 2:**
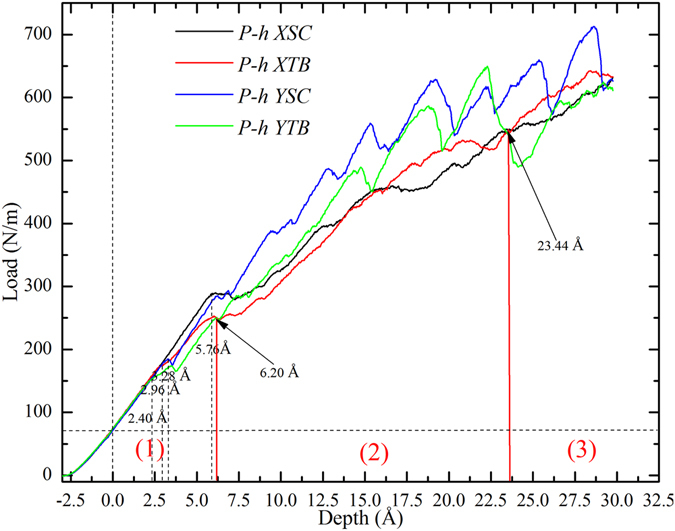
Indentation load-depth (*P-h*) curves of four samples.

The indentation force *F* is calculated by the interaction between two groups of atoms: the indenter and the substrate atoms, hence, there are three components of the tensor are ordered *x, y* and *z*. The indentation force *F* is the component in *z* direction. Due to the different lengths (6 [112] *a* for XSC and XTB, and 8 $[\bar{1}10]$ *a* for YSC and YTB) of the films in the directions of the longitudinal axis of the indenter, it is commonly normalized to the indentation load *P* with

2 $$P=\frac{F}{L},$$

where *L* is the length of the cylindrical indenter. The load-depth (*P-h*) curves are shown in Fig. 2, where the load is positive rather than zero when *h* = 0, as in the work by others^46, 47^, which can be attributed to the repulsion between the indenter and the specimen atoms when their distance becomes smaller than the equilibrium one (*r*_*c*_ = 2.68 Å in Fig. 2) if using a “real” indenter rather than using a fictitious one described by a repulsive potential^48^. At the initial stage, the force *F* of the Hertz solution of for the contact between a rigid cylindrical indenter and an elastic plane can be expressed as^24^:

3 $$F=\frac{\pi {a}^{2}GL}{2(1-\nu )R},$$

In which *a*, *G*, *ν* and *R* are contact half-width, shear modulus of the film, Poisson’s ratio of the film and the radius of the indenter, respectively. The contact half-width *a* can be determined with

4 $$a=\sqrt{{R}^{2}-{(R-{h}_{c})}^{2}},$$

where *h*_c_ is the contact depth. The contact depth in elastic deformation stage can be calculated with

5 $${h}_{c}=1/2h,$$

Substituting Eq. (5) into Eq. (4) and then substituting Eq. (4) into Eq. (3), *F* can be expressed as

6 $$F=\frac{\pi GL}{2(1-\nu )R}(Rh-\frac{{h}^{2}}{4}).$$

Noticing that *h* is much smaller than *R*, *F* can be further simplified as

7 $$F\approx \frac{\pi GL}{2(1-\nu )}h,$$

Hence, the load can be expressed as:

8 $$P=\frac{F}{L}\approx \frac{\pi G}{2(1-\nu )}h.$$

*P* should be approximately proportional to depth *h*, confirmed in Fig. 2, showing the validation of the nanoindentation model. It can be seen that the four curves match well with each other at initial stage before *h* = 2.40 Å, therefore, *G*/(1 − *ν)* is a constant for different samples, indicating that the direction of cylindrical indenter axis have insignificant effect on elastic deformation. However, some differences between these curves become more obvious with the increase of *h*, which will be analyzed from the in-plane anisotropy and the effects of TB.

### In-plane anisotropy in VN(111)

**Figure 3:**
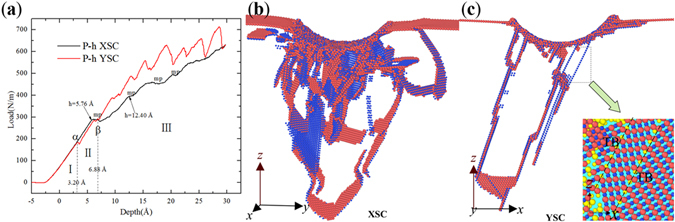
In-plane anisotropy of single crystal VN(111) with cylindrical indenter along different directions. (**a**) *P*-*h* curves of XSC and YSC; (**b**) and (**c**) microstructures in XSC and YSC at *h* = 25 Å, colored with atomic type. The inset is twin structure on *x-z* plane, colored with *CSP*. Atoms with *CSP* < 0.5 and indenter atoms have been removed.

Figure 3(a) shows the *P-h* curves of the samples XSC <112> and YSC <110>, where one can see that these curves can be divided into three parts (I, II and III) by Points *α* (*h* = 3.20 Å) and *β* (*h* = 6.88 Å). In part I, the curves of the two samples match each other. In part II, the curve of XSC is higher than that of YSC, and the curve of XSC becomes lower than that of YSC in the following part. Figure 3(b) and (c) present the microstructures of XSC and YSC at *h* = 25 Å, where the defects in XSC are more obvious than that in YSC. It can be found that the stacking faults (SFs) contain two or three layers of atoms in both samples (Fig. 3(b) and (c)), however, only few TBs form in YSC (Fig. 3(c)). The forming and thickening mechanisms of the TBs are the sequential nucleation and propagation of partial dislocations on adjacent parallel {111} planes, which has been discussed in details in our previous work^19^. A brief description is given as follows, (1) At Point α, the resolved shear stress reaches the critical value, and a partial dislocation (PD) nucleates to relax the internal stress, resulting in a drop near Point α; (2) During the subsequent loading in part II, the movement of the PD and the expansion of the stacking fault become dominant, and the curve of YSC increases with the slope almost the same as in the initial elastic stage; (3) With the further increase of *h*, the local resolved shear stress reaches the critical value again, resulting in the nucleation of a new PD. The twin boundary begins to form with the movement of this new PD, if the new PD nucleates adjacent to the previous nucleated SF. In the subsequent loading, some PDs adjacent to the embryos of twin nucleate, contributing to the thickening of TB, or some PDs nucleate and move, forming some isolated SFs.

**Figure 4:**
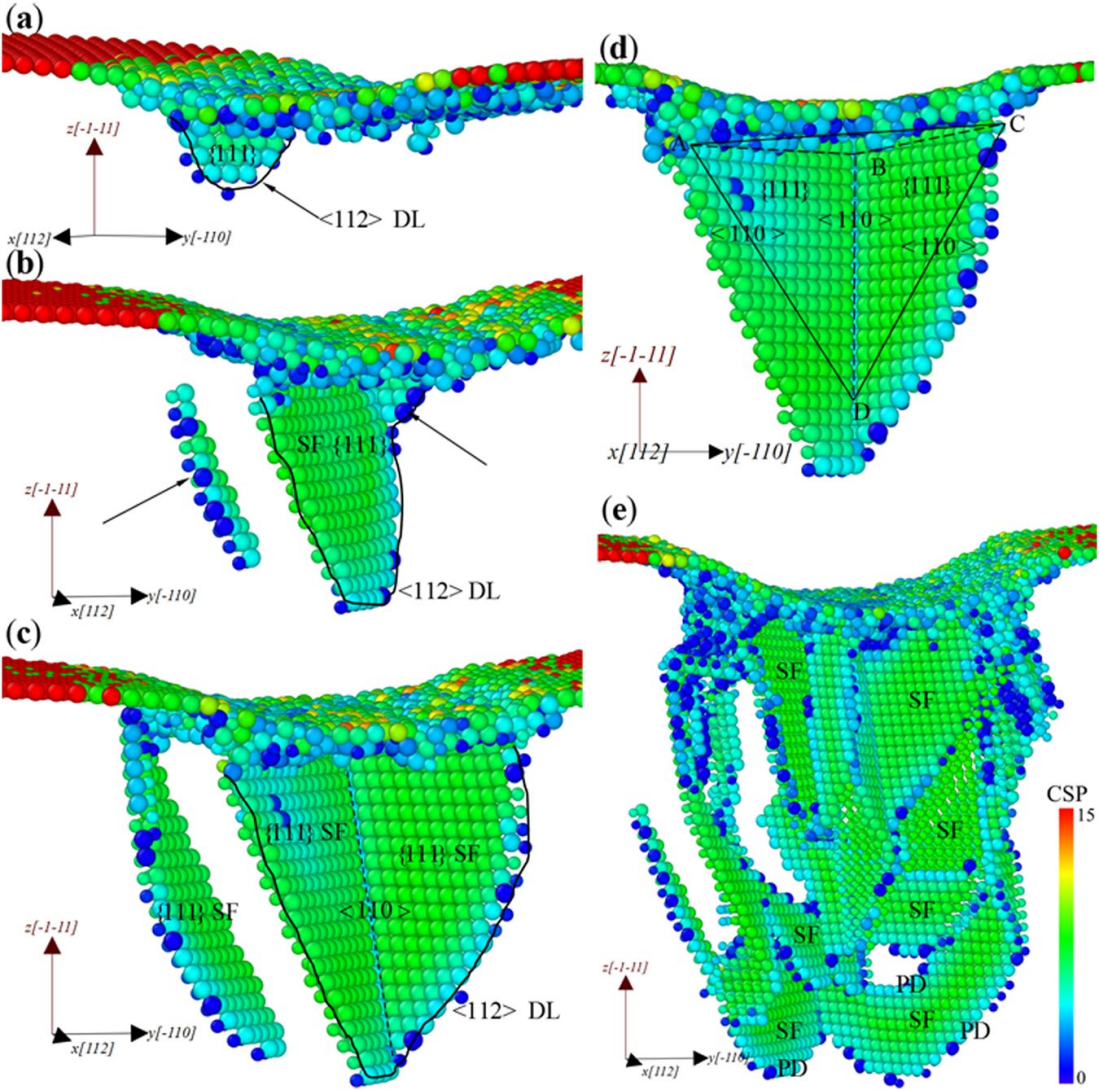
Microstructures of XSC, colored with *CPS*. (**a**) *h* = 5.76 Å, (**b**) *h* = 6.24 Å, (**c**) *h* = 6.72 Å, (**d**) *h* = 6.72 Å for illustration of formation mechanisms of a symmetrical structure formed in XSC and (**e**) *h* = 13.44 Å. Atoms with *CSP* < 0.5 and indenter atoms have been removed.

**Figure 5:**
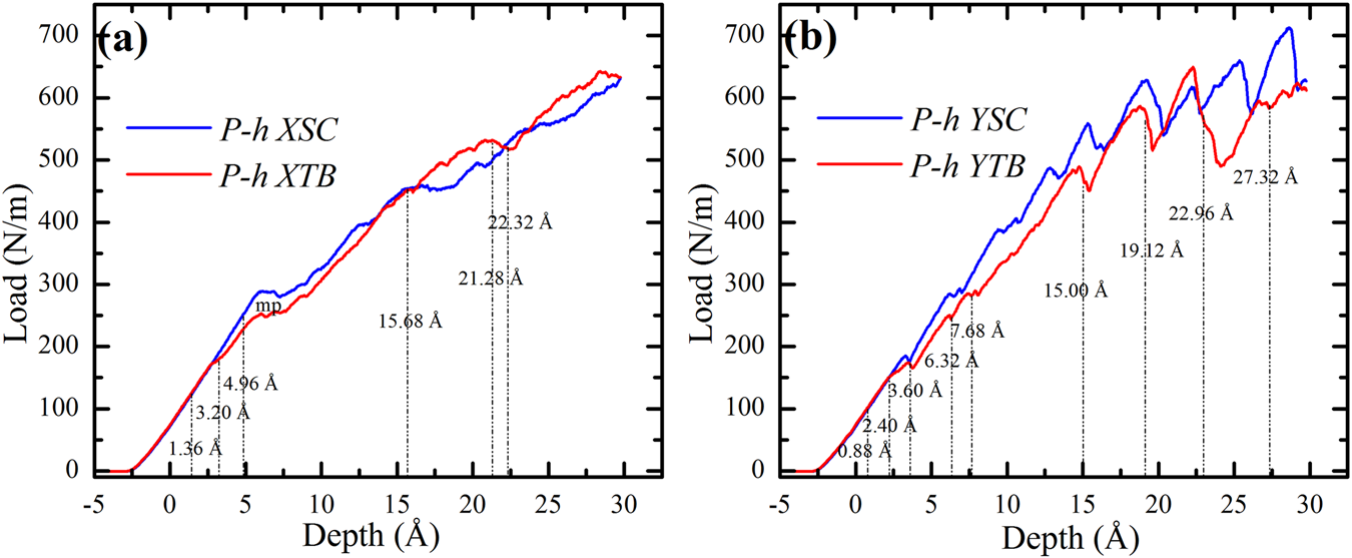
Comparison between *P-h* curves of samples with and without TB. (**a**) Indenter along *x* <112> direction and (**b**) Indenter along *y* <11**0**> direction.

Unlike the zigzag increase in the *P-h* curve of YSC, there is no sharp drop but some minor platforms (labeled with “mp”) can be observed in the *P-h* curve of XSC, as shown in Fig. 3(a), which has also been reported in the nanoindentation simulations with a spherical indenter^38, 49^ or in the case involving large deformation with a cylindrical indenter^18^. To understand the mechanism for these minor platforms, the microstructures of XSC at different depths are shown in Fig. 4. Figure 4(a) shows the microstructure at *h* = 5.76 Å, where one can see that a <112> PD nucleates and moves, forming a {111} SF. With the increase of *h*, more PDs nucleate and glide, resulting in the expansion of the SF and the dislocation loop (DL) in Fig. 4(b). Meanwhile, some new PDs nucleate, forming a {111} SF in parallel with or intersecting with the SF mentioned in Fig. 4(a). Subsequently, the SF, which intersects with the previously formed one, expands with the increase of *h*, forming a structure in symmetry with the *x-z* plane across the central axis of the cylindrical indenter (Fig. 4(c)). Figure 4(d) shows the symmetrical structure observed in Fig. 4(c), where it can be seen that the two SFs are on the {111} planes. Inspired by the result obtained in the nanoindentaion on Au (111)^50^, it can be suggested that dislocations may nucleate on three possible planes (ABD, BCD and ACD) during the nanoindentation on {111} plane. The three planes and the upper surface of the film form a Thompson tetrahedron proposed for FCC structure. Here, the cylindrical indenter is along [112], and the planes ABD and BCD are equivalently the easiest glide planes. Therefore, a symmetrical structure forms when *h* reaches a critical value. This kind of structure is not found in the nanoindentation on YSC. For YSC, the easiest glide plane is the plane ACD when the cylindrical indenter is along $[\bar{1}10]$ direction, and the SFs in YSC are almost parallel (Fig. 4(c)). Back to the minor platforms in Fig. 4(a), the main deformation mechanisms are the nucleation of dislocation, the expansion of stacking faults and dislocation loop in XSC (Fig. 5(a–c)). Therefore, the minor platforms may be ascribed to multiple nucleation of dislocations during the expansion of SFs and the DL, as was reported in metals^23^. Figure 4(e) shows the microstructures of the sample XSC at the second minor platform, where more dislocations nucleate and more SFs and DLs form. On the whole, because the dislocations can nucleate and glide on the two easiest glide planes to release the internal stress, few TB form in the sample XSC. But for YSC, there is only one easiest glide plane for the relaxation of internal stress. Hence, if the some PDs nucleate and glide adjacent to the preformed SF, TB may form and thicken^19, 51^. Therefore, the reason that the *P-h* curve of XSC is lower than that of YSC should be attributed to the different deformation mechanisms induced by the indenter directions. The main deformation mechanism for the indenter direction along <112> (XSC and XTB) is dislocation nucleation and glide. However, the main deformation mechanism for the indenter along <110> (YSC and YTB) is twinning.

### Effects of TB on hardness

Figure 5 shows the comparison between the *P-h* curves of the samples with and without TB, where it can be seen that the *P-h* curve of XTB is higher than that of XSC when *h* > 16.52 Å, indicating the strengthening induced by TB. However, the curve of YTB is generally lower than that of YSC, implying the softening effects of TB. These interesting results can be attributed to different deformation mechanisms, twinning and dislocation gliding, for nanoindentation with cylindrical indenter along different directions.

**Figure 6:**
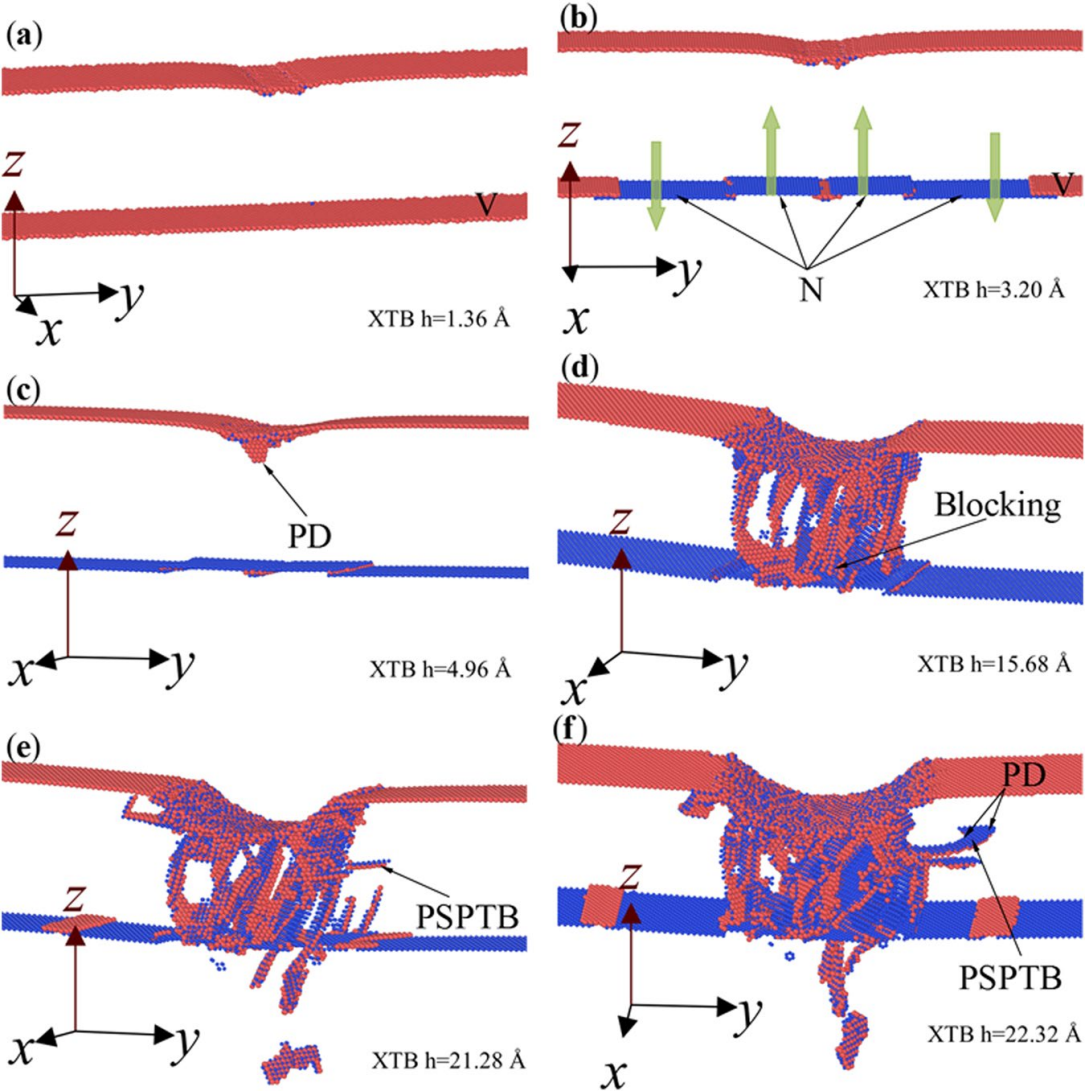
Microstructures evolution in XTB colored with atomic type at different depths. (**a**) *h* = 1.36 Å, (**b**) *h* = 3.20 Å, (**c**) *h* = 4.96 Å, (**d**) *h* = 15.68 Å, (**e**) *h* = 21.28 Å and *h* = 22.32 Å. Atoms with *CSP* < 0.5 and indenter atoms have been removed.

Figure 6 shows the microstructures in XTB at different *h*. By comparing Fig. 6(a) and (b), it can be seen that the TB moves upper or down perpendicular to the TB, from V atoms to N atoms, which contributes to the decrease of the first yield depth. During the movement of TB, some PDs nucleate and glide, forming SF parallel to the TB and relaxing the internal stress, accounting for the lower *P-h* curve of XTB when *h* < 4.96 Å. At *h* = 4.96, a new PD nucleates beneath the indenter (Fig. 6(c)) resulting in the second softening. Then many PDs nucleate simultaneously, leading to the further relaxation of the internal stress, and resulting in an “mp” in the *P-h* curve of XTB. In the following region, the *P-h* curve of XTB ascends persistently, catches up with and even over the *P-h* curve of XSC, exhibiting strengthening effect of TB. Figure 6(d) shows the microstructures of XTB at *h* = 15.68 Å, where one can see that the some dislocations are blocked by the TB, leading to the enhancement of the strength. A slight drop occurs at *h* = 21.28 Å, and the corresponding microstructure is shown in Fig. 6(e). It can be seen in Fig. 6(e) that some PDs nucleate and glide, forming a partial slip parallel with the twin boundary (PSPTB), which may results in softening, and the softening stops as the PSPTB moves away, as shown in Fig. 6(f) when *h* = 22.32 Å. Then the dislocation blocking induced strengthening dominates the linear increase in the following part of the *P-h* curve.

**Figure 7:**
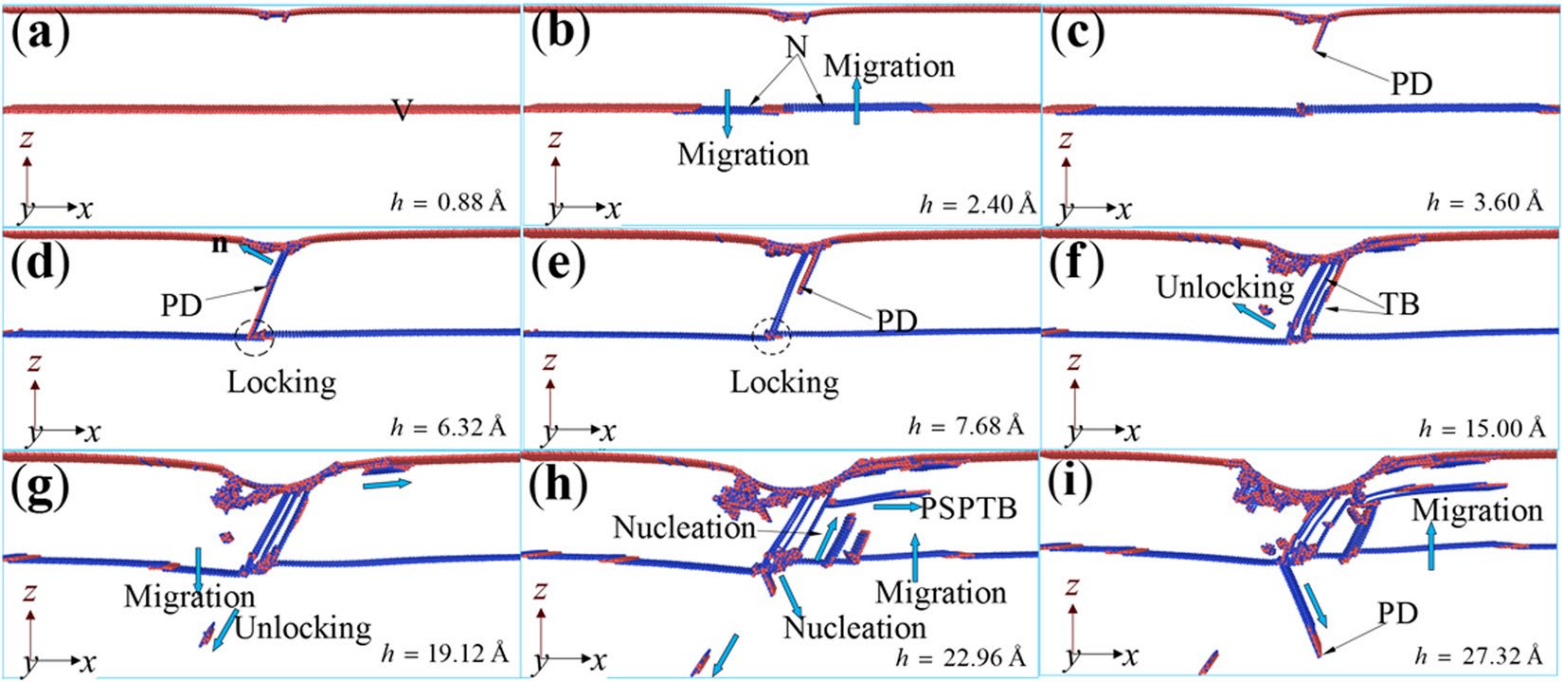
Microstructures evolution of YTB colored with atomic type at different depth. (**a**) *h* = 0.88 Å, (**b**) *h* = 2.40 Å, (**c**) *h* = 3.60 Å, (**d**) *h* = 6.32 Å, (**e**) *h* = 7.68 Å, (**f**) *h* = 15.00 Å, (**g**) *h* = 19.12 Å, (**h**) *h* = 22.96 Å, and (**i**) *h* = 27.32 Å.

Figure 7 gives the evolution of the microstructures in YTB at different *h*. Similar to the results of XTB, the migration of TB leads to softening by comparing the result in Fig. 7(a) with that in Fig. 7(b). With the increase of *h*, the nucleation of PD beneath of indenter (Fig. 7(c)) may occur as the resolved shear stress reaches the critical value, corresponding to the second yield point in the *P-h* curve of YTB (Fig. 5(b)). With the increase of *h*, the PD in Fig. 7(c) glides to reacts with the TB (Fig. 7(d)). However, the main reason for the drop at *h* = 6.32 Å should be the nucleation of a new PD (Fig. 5(b)), contributing to the thickening of TB along the direction of n (Fig. 7(d)). It is known that the sequential nucleation and propagation of PD on the adjacent parallel {111} planes should be the key to the thickening of deformation twin^19^, but from the discussion in the previous subsection the new PD can also nucleate on adjacent or away from the preformed SF. Figure 7(e) shows a new PD nucleating away from the preformed SF in Fig. 7(d), which results in the softening near *h* = 7.68 Å (Fig. 5(b)). It should be noted that the SF may be locked by TB (see the ellipse in Fig. 7(d) and (e)), which may be some kind of strengthening factor but cannot play a dominant role. During 7.68 Å < *h* < 14.65 Å, the *P-h* curve of YTB develops linearly with several slight fluctuations, corresponding to softening induced by the nucleation of PDs, forming TB or unlocking of the dislocation from the locked site. Figure 7(g–i) show the microstructures of YTB at the three drop points, *h* = 19.12 Å, 22.96 Å, and 27.32 Å, respectively, where one can see that the softening can be attribute to the migration of TB, the unlocking and nucleation of dislocations, and PSPTB, which can release the internal stress. Therefore, we can conclude that softening mechanism should be responsible for the lower *P-h* curve of YTB (Fig. 5(b)).

In Fig. 2, the comparison between the *P-h* curves of XTB and YTB can be divided into three segments (1), (2) and (3) by *h* = 6.20 Å and *h* = 23.44 Å. To understand these curves, we introduce a simple expression

9 $$P={P}_{0}+{P}_{{\rm{strengthen}}}-{P}_{{\rm{soften}}}$$

In which *P*_strengthen_ and *P*_soften_ correspond to the contributions from strengthening and softening effects, respectively. *P*_0_ is the load without taking into account the strengthening and softening effects. In Segment (1), PD nucleates firstly in YTB due to the smaller critical stress, which contributes to *P*_*soften*_ and results in the lower *P-h* curve of YTB. In Segment (2), the PDs and SFs in XTB are much more obvious than that in YTB, which contributes to *P*_*soften*_ and results in the lower *P-h* curve of XTB, though a little strengthening effect due to dislocations blocked by TB appears. With the further increase of *h*, the dislocation blocking induced strengthening begins to play a dominant role in XTB and this effect is much stronger than that in YTB, because a larger number of PDs have nucleated in Segment (2) in XTB than in YTB. On the other hand, the deformation mechanisms including the migration of TB, the unlocking and nucleation of dislocation, and PSPTB due to the concentration of internal stress, induce softening in YTB. With the effect of the two aspects, the *P*-*h* curve of XTB is higher than that of YTB in Segment (3).

To guarantee the validity of the results, additional nanoindentation simulations are performed on the four samples with a lower indentation speed of 20 m/s and at higher temperature of 300 K, and similar result have been obtained, of which the indentation *P*-*h* curves and the microstructures at some typical points are shown in Figures S2–S4 in Supplementary Materials.

## Conclusions

Nanoindetations on VN (111) films with or without twin boundary were performed with molecular dynamics simulations, in which cylindrical indenters along directions <112> and <110> were used to investigate the in-plane anisotropy and the effects of TB. It was found that the direction of the cylindrical indenter has insignificant effect in elastic stage, but it becomes significant in the following inelastic deformation stage. The strengthening or softening mechanisms involve twinning and dislocation glide, which were analyzed by a Thompson tetrahedron. We found that the internal stress can be released by the migration of TB, which results in softening; while the dislocation locking and pile-up at TB can enhance the strength.

However, the strengthening or softening by TB may be related to the many factors, such as the twin thickness, temperature, loading parameters (indentation speed, indenter size), etc. These results are the basis for the study of twin thickness effect and the effect of twinning in polycrystalline transition metal nitrides.

## Electronic supplementary material

Supplementary Information
