# Supplementary material for: In-plane anisotropy and twin boundary effects in vanadium nitride under nanoindentation
Source: Sci Rep. 2017 Jul 6;7:4768. doi: 10.1038/s41598-017-05062-0 (PMC5500517; doi:10.1038/s41598-017-05062-0)
Supplement: Supplementary file 1 — Supplementary Information [file 41598_2017_5062_MOESM1_ESM.doc]

**Supplementary Materials**

**In-plane anisotropy and twin boundary effects in vanadium nitride under nanoindentation**

Tao Fu ^1^, Xianghe Peng[[1]](#footnote-2), 2, *, Cheng Huang ^1^, Henggao Xiang ^1^,

Shayuan Weng ^1^, Zhongchang Wang ^1, 3^, Ning Hu ^1, *^

*^1^Department of Engineering Mechanics, Chongqing University, Chongqing 400044, China*

*^2^ State Key Laboratory of Coal Mine Disaster Dynamics and Control, Chongqing University, Chongqing 400044, China*

*^3^International Iberian Nanotechnology Laboratory (INL), Avenida Mestre Jose Veiga Braga 4715-330, Portugal*

**1 The comparison between the generalized stacking fault energy (GSFE) and twin fault energy (TFE) curves**

We calculate GSFE and TFE curves with the formula $\gamma(\mathbf{d})=\frac{E_{\mathbf{d}}-E_{0}}{A}$, where E_0_ and E_d_ and *A* are the potential energies of the system before and after being subjected to a relative displacement **d**, *A* is the stacking-fault area, as shown in Figure S1, where one can see that the γ_TFE_ is lower than γ_SF_.

Figure S1. The comparison between the GSFE and TFE curves.

**2. Nanoindentation simulations with indentation speed of 20 m/s at 300 K**

Figure S2 shows the P-h curves of samples with indentation speed of 20 m/s at 300 K. The fluctuations in the curves are obvious larger than that at 10 K in Fig. 2. However, the results obtained at 300 K can in principle support the conclusions obtained at 10 K.

(1) The curves of sample obtained at 300 K match well with each other at initial elastic stage, showing that the direction of cylindrical indenter axis have an insignificant effect on elastic deformation.

(2) The curve of YSC is obvious higher than that of XSC, which corresponds to different deformation mechanisms, twinning and dislocation glide. (Fig. S3)

(3) Both the migrations of TB are observed in XTB and YTB at these simulations due to the complex of the nanoindentation. (Fig. S4)


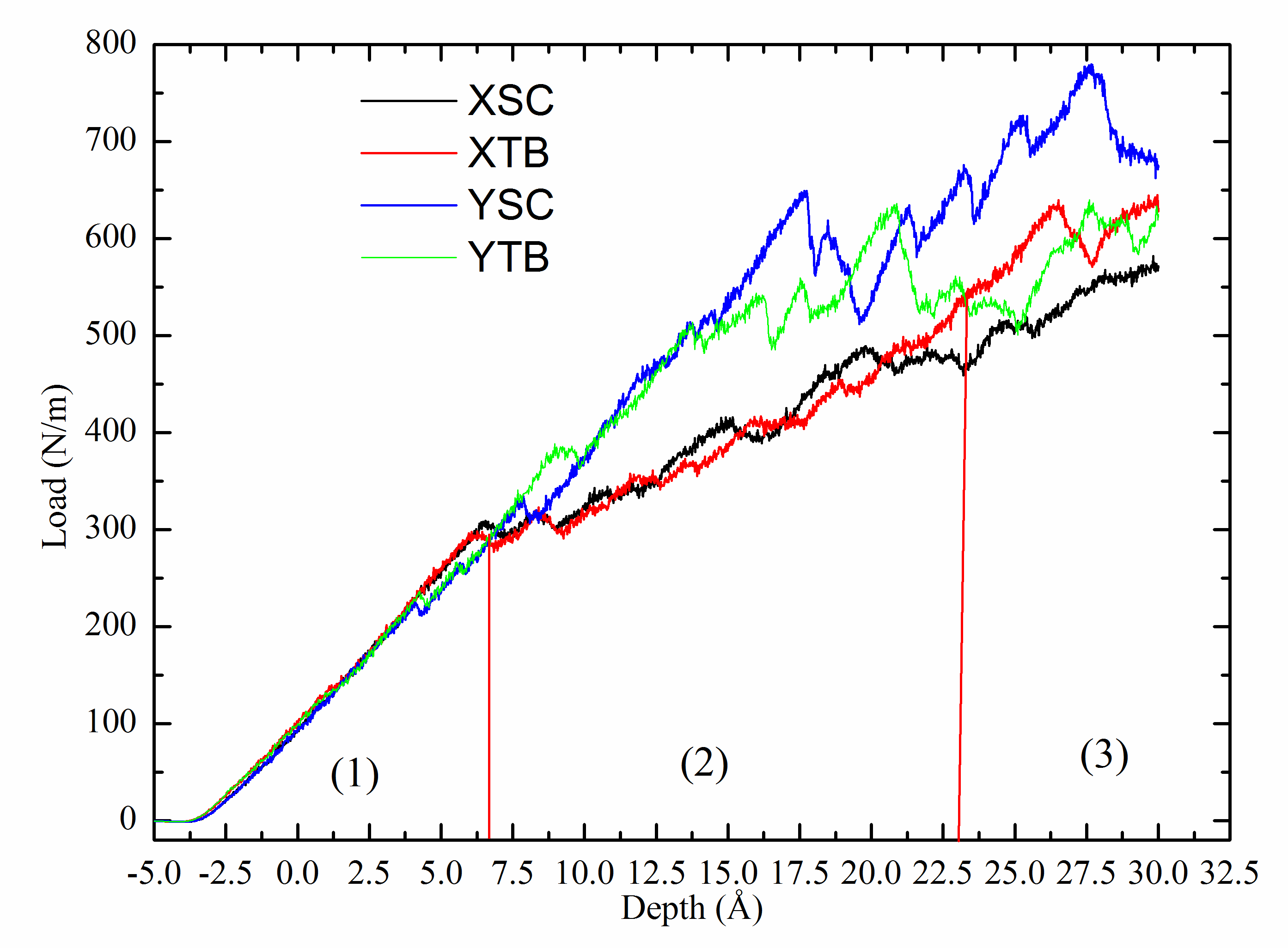


**Figure S2.** Indentation load-depth (*P-h*) curves of four samples with indentation speed of 20 m/s at 300 K.


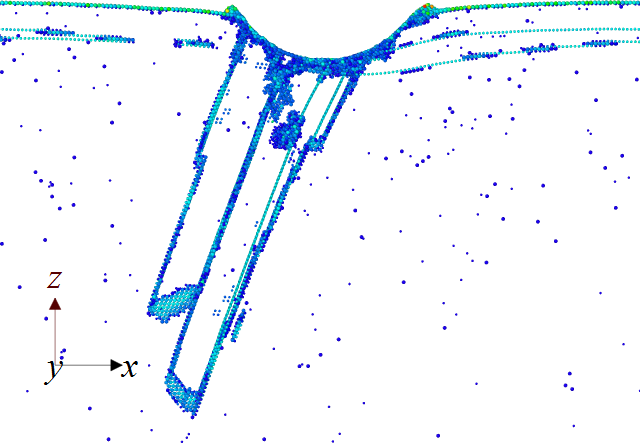

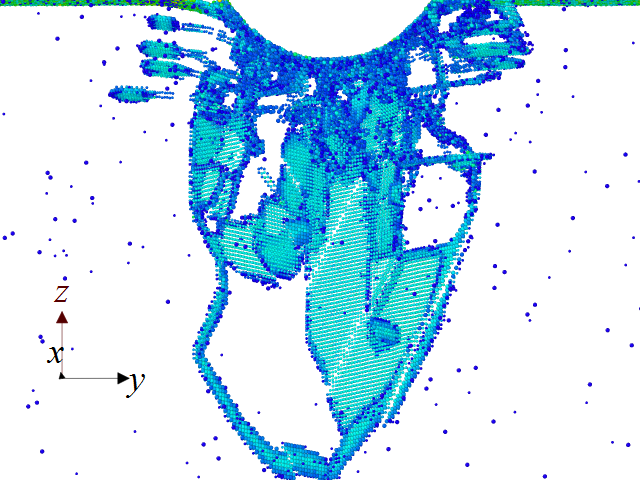


**Figure S3.** In-plane anisotropy of single crystal VN(111) with cylindrical indenter along different directions. (a) <112> and (b) 110 directions.


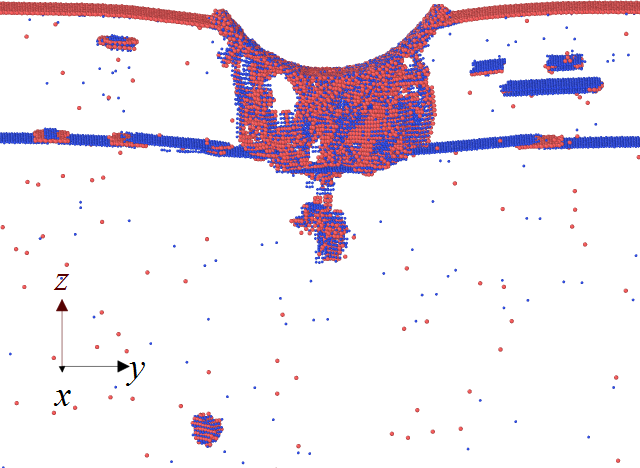

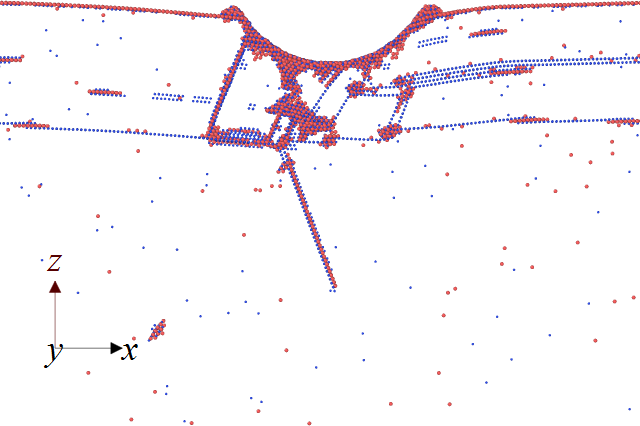


**Figure S4.** Comparison of the microstructure between XTB and YTB.

1. *Corresponding authors.

   Address: College of Aerospace Engineering, Chongqing University, Chongqing 400044,China

   TEL: +86–23–65103755; FAX: +86–23–65102521

   E-mails: xhpeng@cqu.edu.cn (X.P.); ninghu@cqu.edu.cn (N.H.). [↑](#footnote-ref-2)
